# PReS-FINAL-2221: An earliest diagnosis of FMF

**DOI:** 10.1186/1546-0096-11-S2-P211

**Published:** 2013-12-05

**Authors:** G Keskindemirci, N Aktay Ayaz, E Aldemir, C Aydogmus, G Aydogan, S Kavuncuoglu

**Affiliations:** 1 Kanuni Sultan Suleyman Education and Research Hospital, Istanbul, Turkey

## Introduction

Familial Mediterranean fever (FMF) is an autosomal recessive disease, mainly affecting Jews, Armenians, Turks, Arabs and other groups living around Mediterranean basin. Major symptoms of disease are recurrent periodic fever accompained by serositis. The disease is usually diagnosed at ages less than 20 years. Onset of the disease atolder age can rarely occur. Symptoms related to FMF are noted when children become more verbal, usually after 2 years of age. Mutation analysis supports diagnostic evaluation.

## Objectives

Here, we are reporting the youngest FMF patient, that were internalized after birth as sepsis. Physicians were unable to discharge her from the hospital due to high acute phase response, that was dedicated to meningitis, urinary tract infection, sepsis and so on. Her metabolic screenings were done and were found to be negative. She was consulted to pediatric rheumatology for the high acute phase response and fever. With a detailed history and evaluation, it was learned that her mother had recurrent swelling of her ankle joints. Mutation analysis was performed and two homozygous mutations (M694V andR202Q) were identified. She was diagnosed as FMF at 3 months of age and colchicine was started with a dose of 0.25 mg/day. She responded to colchicine both clinically and in laboratory basis. Her uncontrolled acute phase response declined gradually.

## Conclusion

This case was reported to point out the importance of early remembrance of possible autoinflammatory diseases even at very early ages especially at endemic countries.

## Disclosure of interest

None declared.

